# A deep learning generative model approach for image synthesis of plant leaves

**DOI:** 10.1371/journal.pone.0276972

**Published:** 2022-11-18

**Authors:** Alessandro Benfenati, Davide Bolzi, Paola Causin, Roberto Oberti

**Affiliations:** 1 Dept. of Environmental Science and Policy, Università degli Studi di Milano, Milano, Italy; 2 Dept. of Mathematics, Università degli Studi di Milano, Milano, Italy; 3 Dept. of Agricultural and Environmental Sciences—Production, Landscape, Agroenergy, Università degli Studi di Milano, Milano, Italy; Institut de Robotica i Informatica Industrial, SPAIN

## Abstract

**Objectives:**

A well-known drawback to the implementation of Convolutional Neural Networks (CNNs) for image-recognition is the intensive annotation effort for large enough training dataset, that can become prohibitive in several applications. In this study we focus on applications in the agricultural domain and we implement Deep Learning (DL) techniques for the automatic generation of meaningful synthetic images of plant leaves, which can be used as a virtually unlimited dataset to train or validate specialized CNN models or other image-recognition algorithms.

**Methods:**

Following an approach based on DL generative models, we introduce a Leaf-to-Leaf Translation (L2L) algorithm, able to produce collections of novel synthetic images in two steps: first, a residual variational autoencoder architecture is used to generate novel synthetic leaf skeletons geometry, starting from binarized skeletons obtained from real leaf images. Second, a translation via Pix2pix framework based on conditional generator adversarial networks (cGANs) reproduces the color distribution of the leaf surface, by preserving the underneath venation pattern and leaf shape.

**Results:**

The L2L algorithm generates synthetic images of leaves with meaningful and realistic appearance, indicating that it can significantly contribute to expand a small dataset of real images. The performance was assessed qualitatively and quantitatively, by employing a DL anomaly detection strategy which quantifies the anomaly degree of synthetic leaves with respect to real samples. Finally, as an illustrative example, the proposed L2L algorithm was used for generating a set of synthetic images of healthy end diseased cucumber leaves aimed at training a CNN model for automatic detection of disease symptoms.

**Conclusions:**

Generative DL approaches have the potential to be a new paradigm to provide low-cost meaningful synthetic samples. Our focus was to dispose of synthetic leaves images for smart agriculture applications but, more in general, they can serve for all computer-aided applications which require the representation of vegetation. The present L2L approach represents a step towards this goal, being able to generate synthetic samples with a relevant qualitative and quantitative resemblance to real leaves.

## Introduction

The ability to generate realistic synthetic images of leaves has been explored since time in the field of computer graphics to create scenes with credible landscapes covered with plants, trees or meadows for use in computer games, virtual reality and entertainment industry (*e.g.*, see the review in [1] by authors from Disney Research Studios) or in architectural rendering [2]. Current interest has been expanding to quantitative applications related to advanced approaches in botanics, plant breeding and agriculture, including morphometric analysis of plants or organs, phenotyping, simulation of light distribution in canopies, biochemical and photosynthesis modeling, growth analysis and response of plants to treatments, study of spraying deposit on leaves (see [3] for a comprehensive review of these applications).

In literature, the methods developed for algorithmic generation of the geometry of digital models of leaves can be regrouped in three approaches: *i)* rule–based; *ii)* model–based; *iii)* image/point cloud–based. Methods of class *i)* rely on abstract mathematical procedures encoded in a set of rules, with output depending on parameters assigned by the user. For example, Peyrat et al [4] applied the so–called L–grammar [5] to create different instances of leaf silhouettes and veins, simulating intra-class variability by random fluctuations of the used parameters. This approach, solely based on a topological rules, was able to produce thousands of different synthetic leaf images, yet completely uncorrelated to existing leaves. Methods of class *ii)* consider models built upon biological hypotheses. The main idea is that leaf veins and margins determine the characteristics of the leaf blade during the process of leaf formation, which occurs on the basis of biochemical signals. In this context, Runions et al [6] proposed a leaf venation model driven by the spatial interaction of auxin hormone sources distributed over a surface -to become the leaf blade- and the formation of vein patterns. Alsweis et al [7] regarded leaf tissue as a viscous, incompressible fluid whose 2D expansion was determined by a spatially varying growth rate that reacted to auxin sources embedded in the leaf blade. These biophysically–based models require a complex fine-tuning of several parameters in order to produce realistic results, with limited flexibility in reproducing synthetic leaves in different states of their life–cycle. Methods in class *iii)* aim at reproducing the geometry of a specific plant/canopy from physical measurements or from images. In this context, Quan et al [8] used a hand-held camera to capture images of a plant from different viewpoints. Upon registration and processing of the acquired images, they obtained a 3D point cloud from which an editable geometric model of single leaves was extracted. Tang et al [9] defined key points on the leaf edge by user interaction on 2D images and from them a triangular mesh of the leaf was constructed and finally shaped in 3D by applying deformations driven by a mass-spring model of the leaf. Gèlard et al [10] developed an algorithm to segment and label stereo-images of different leaves from sunflower plants. Parametric surface modeling via NURBS representation was adopted to analytically describe the leaf geometry, enabling to implement a flexible model to quantify plant growth from automatic measure from sensors. Whilst able to generate accurate editable geometrical models of a specific plant, a major lack of these image/point cloud–based approaches is that they are not capable to produce large and varied collections of different leaves. Irrespective of the type of approach used in the above classes, an interesting point is that leaf blade texture and color were always rendered in a separate step by means of a color palette defined by the user or generated according to a certain algorithm. Colorization can thus be considered as a separate problem addressed once the leaf geometry is available, and is typically implemented via an *ad hoc* third–party rendering software. Among the various approaches to obtain the color map to be used, we cite here the color model based on convolution sums of divisor functions proposed in [11], the shading model based on the PROSPECT model for light transmission in leaves [12] of [13], the “virtual rice” leaves generated in [14] from a RGB–SPAD color model and the Markov chain model of [10] in which environmental factors as air temperature and soil moisture were used as conditions to drive the probability to transfer from one state (texture and color) to another of the Markov chain.

In this work we propose an approach, at the best of our knowledge not explored before (see [15] for a state–of–the–art review), and radically different from those cited above. Our aim is to introduce and implement Deep Learning (DL) techniques to automatically generate collections of synthetic images of plant leaves. These virtually unlimited synthetic images are then to be employed in our case study to enrich dataset of natural leaf images used for training Convolutional Neural Networks (CNN) dedicated to smart agriculture applications. CNN models require indeed enormous amounts of annotated training images examples to avoid overfitting phenomena. Yet in real world applications annotated data are very often limited, especially for semantic segmentation tasks that typically require pixel-scale accuracy in manual labeling of training images. Even standard image augmentation methods, usually consisting in simple color and geometric transformations such as random rotations, translations, scaling or deformations of the original images, provide limited richness of the augmented dataset. DL generative models represent attractive methods to produce large sets of synthetic images (with corresponding labels), starting using from the information from a limited set of natural (*i.e.*, real), unlabeled images of the same domain. This approach has recently emerged in medical imaging research, where data may be extremely scarce and difficult to obtain (*e.g.*, see the recent review [16]). For our model we take inspiration from [17] (and the research referenced therein), where the authors synthesized eye retina images. Indeed, the fundus of the eye shares several characteristics with our problem: a fine network of hierarchically organized blood vessels (as the leaf veins) superposed to a colored background (as the tissue of the leaf blade). Unlike to [17], in our problem the leaf blade has a specific shape that must be also meaningfully generated in the synthetic image. We call the algorithm proposed hereby L2L, *i.e.* a Leaf-to-Leaf Translation approach to obtain synthetic colorized leaf images. The L2L algorithm is organized in two steps described in the following sections. First it uses a residual variational autoencoder architecture to generate novel (unreal) leaf skeletons starting from binarized companion skeletons of real leaf images. Second, it performs a translation via Pix2pix framework, which uses conditional generator adversarial networks (cGANs) to reproduce the specific color distribution of the leaf blade, preserving leaf shape and venation pattern. To evaluate the obtained results, we then present both qualitative and quantitative evaluations of the degree of realism reached by the generated synthetic leaf images. For this, a DL-based anomaly detection strategy is used to evaluate the distance (“anomaly”) between synthetic and real images. Eventually, as an illustrative example of application of the proposed method, we detail the implementation of L2L algorithm for generating a set of images aimed at training a CNN model for automatic detection of disease symptoms in cucumber leaves.

## Materials and methods

### Dataset

Fresh grapevine leaves from the greenhouses of the Department of Agricultural and Environmental Sciences of University of Milano were collected and imaged via a QSi640 ws-Multispectral camera (Atik Cameras, UK) equipped with a Kodak 4.2 Mp micro-lens image sensor and 8 pass–band filters operating from 430 to 740 nm. For the purpose of this experiment, leaves were imaged singularly on a dark background, under controlled diffuse illumination conditions. Images were acquired in the single spectral channels R, G, B bands (with pass-band filters centered at 685 nm, 530 nm, 430 nm, respectively) and in the near–infrared, NIR (filter centered at 740 nm). NIR channel was included in this study as it is very commonly considered in crop sensing [18, 19]. This is due to a very low absorbance of plant tissue in this band (no photoactive pigments or other compounds absorb light significantly around these wavelengths), which enables accurate foreground segmentation of vegetation in field images, and at leaf scale allows to obtain a homogeneous picture of the leaf blade surface structure. A set of RGB images of the same leaves in standard CIE color space were also acquired for reference. Camera parameters were set and image collection was performed via an *in–house* developed acquisition software written in MATLAB. Reflectance calibration was carried out by including in each image 3 reflectance references (Spectralon R = 0.02, R = 0.50 and R = 0.99; Labsphere, USA). We obtained images of 80 leaves with a resolution of 2048×2048 pixels and 8 bit for each channel. Preprocessing operations were performed on each image:

- removal of *hot* pixels: for each channel and for each pixel, we computed the difference between the pixel value and its neighbors. If all these differences were greater than the 10% of the average between the minimum and maximum of the pixel values, we replaced the pixel with the average of its neighbors
- normalization along each channel: let *m* be the minimum value of the 2% reference probe and *M* the maximum value of the 99% reference probe. Then we set *p* ← (*p*−*m*)/(*M*−*m*), where *p* is the pixel value
- creation of a companion skeleton image, which comprises the profile of the leaf and the vein pattern. This procedure was carried out using the NIR channel, since this presents a high contrast between the leaf and background. First, the image was binarized using the Otsu method, which chooses the threshold value that minimized the intraclass variance of the thresholded black and white pixels. Then the Moore-Neighbor tracing algorithm was used to detect the outer boundary of the leaf and the internal venation pattern
- automatic crop to center the leaf in the image and resizing at 256×256 resolution in for all the channel acquisitions

**Fig 1:**
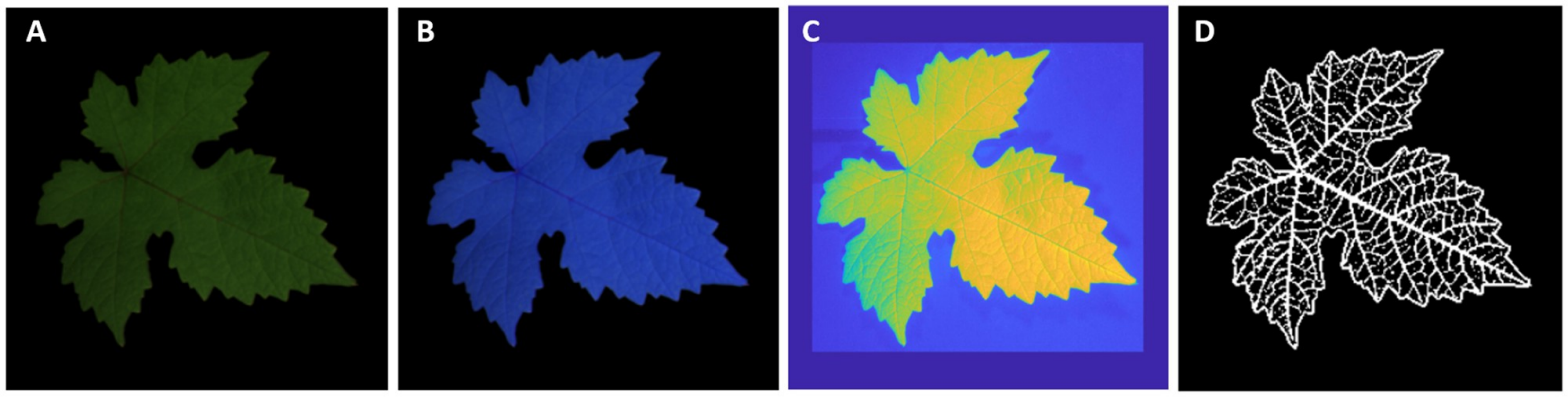
Sample of grapevine leaf from the dataset. A: RGB image; B: RGNIR image; C: normalized and cropped NIR image; D: companion skeleton. In the skeleton binarized image, the white color identifies the leaf profile and veins, the black color identifies other parts of the leaf and the background.

Fig 1 shows a sample of images o the original images in the RGB and RGNIR spaces, the normalized NIR channel and the corresponding companion skeleton. Before using the generative algorithms, we performed standard data augmentation by randomly flipping each image horizontally and vertically, rotating by an angle randomly chosen in [−*π*/4, *π*/4] and finally zooming with a random amount in the range [−20%, + 20%]. The dataset was thus increased in this way from 80 to 240 samples.

### Generative methods for L2L translation

The authors of [17, 20] generated artificial patterns of blood vessels along with corresponding eye fundus images using a common strategy which divides the problem of the image generation into two sub–problems, each one addressed by a tailored DL architecture: first they generate the blood vessel tree, then they color the eye fundus. We adopt this very approach, first generating the leaf profile and veins and then coloring the leaf blade. Also in our experience this approach has turned out to be more effective than generating the synthetic image altogether. We refer to S2 File. for a further discussion about the motivation of the present two-step approach.

#### Skeleton generation

**Fig 2:**
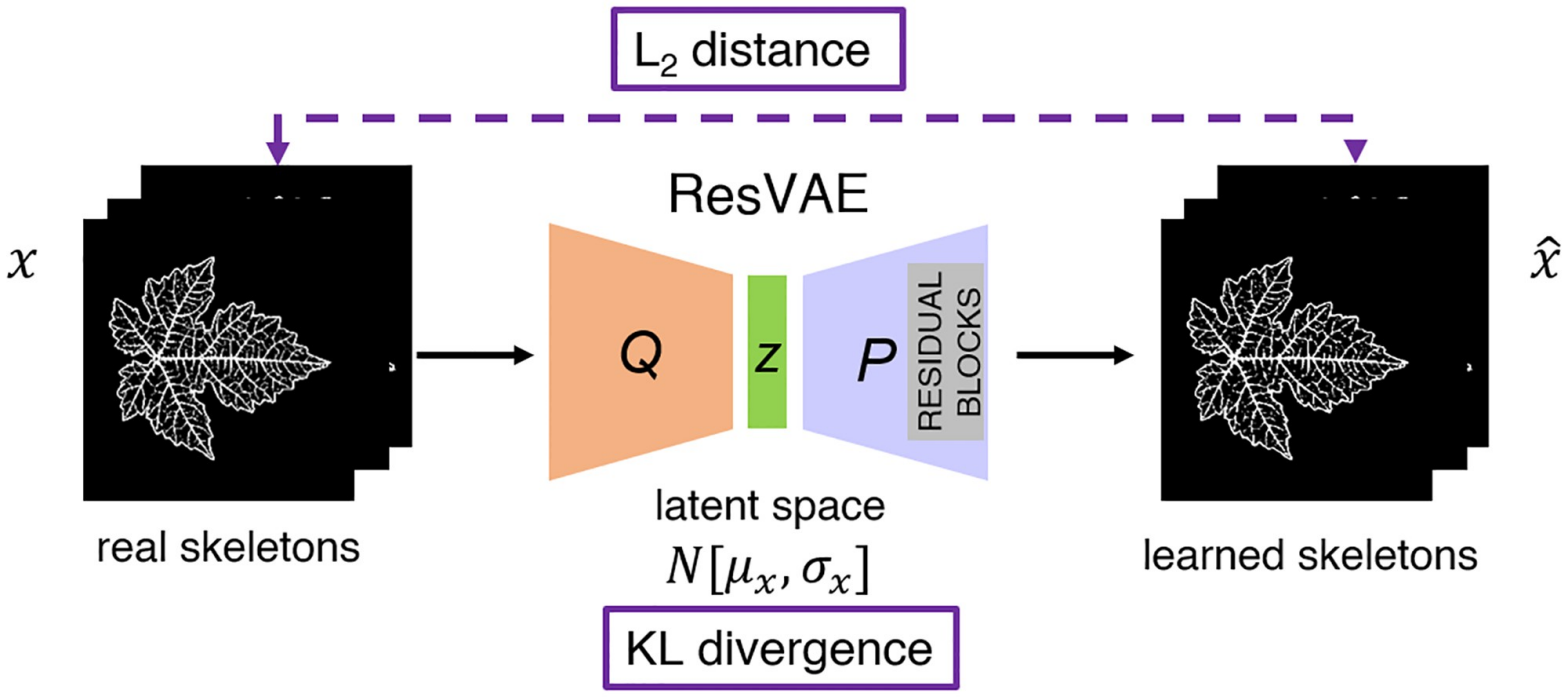
Illustration of the ResVAE architecture (training phase).

According to the above considerations, the generation of a realistic leaf skeleton is the first step towards the final goal of our work. For this task, we use a convolutory autoencoder architecture, that is, a network trained to reconstruct its input. An autoencoder (AE) is composed of two submodels: 1) an encoder *Q* that maps the training dataset to a latent (hidden) representation *z*; 2) a decoder *P* that maps *z* to an output that aims to be a plausible replica of the input. We have experimented that simple autoencoders cannot generate realistic skeletons. For this reason, we use a more sophisticated architecture, called Residual Variational Autoencoder (ResVAE, see Fig 2).

This learning framework has already been successfully applied to image recognition, object detection, and image super-resolution (see, *e.g.*, [21]). In the data generation framework, AEs learn the projection of the initial data into a *latent subspace*, and then a sample of this subspace is randomly extracted to build up a new instance of the initial data. Instead of learning such projection, VAEs learn the probability distribution of the latent variables given the input *x*. As a matter of fact, a variational autoencoder can be defined as an autoencoder whose training is regularized to avoid overfitting and ensure that the latent space has good properties that enable the generative process. To achieve this goal, instead of encoding an input as a single point, VAEs encode it as a (Gaussian) distribution over the latent space, where *p*(*z*|*x*) represents the probability of the latent variable *z* given the input *x*. The decoding part consists in sampling a variable from *p*(*z*|*x*) and then providing a reconstruction $\hat{x}$ of the initial data *x*. We associate to this framework the following loss function

$$\begin{array}{c} L_{\text{VAE}}\left(x,\hat{x}\right)=L_{L_{2}}\left(x,\hat{x}\right)+\beta L_{KL}(p(z|x),N(0,1)), \end{array}$$ (1)

where the first term $L_{L_{2}}=||x-\hat{x}{\left|\right|}^{2}$ is the *L*_2_ norm of the reconstruction loss, and the second term $L_{KL}=KL\left[N\left(\mu_{x},\sigma_{x}\right),N\left(0,1\right)\right]$ is the Kullback–Leibler (KL) divergence [22–24] The KL divergence enhances sparsity in neurons activation to improve the quality of the latent features keeping the corresponding distribution close to the Gaussian distribution N(0,1). The tunable regularization hyperparameter *β* is used to weigh the two contributions [25]. With respect to VAEs, ResVAEs additionally employ residual blocks and connection skips. The idea beyond residual blocks is the following [26]: normal layers try to directly learn an underlying mapping, say *h*(*x*), while residual ones approximate a residual function *r*(*x*) = *h*(*x*) − *x*. Once the learning is complete, *r*(*x*) is added to the input to retrieve the mapping: *h*(*x*) = *r*(*x*) + *x*. In our architecture, residual blocks are concatenated to the decoder to increase the capacity of model [21]. The connection skips allow to back–propagate the gradients more efficiently giving the bottleneck more access to the simpler features extracted earlier in the encoder. The resulting ResVAE compresses 256 × 256 leaf skeleton images to a low dimension latent vector of size 32 and then it reconstructs it to 256 × 256 images. We refer to Section A in S1 File. for specifications of the present ResVAE architecture and the relative training strategy.

#### Translation to colorized leaf image

We consider the colorization of the leaf out of an existing skeleton as an image-to- image translation problem, which implies to learn a mapping from the binary vessel map into another representation. Similarly to what observed in [17] for retinal image generation, many leaf images can share a similar binary skeleton network due to variations in color, texture, illumination. For this reason, learning the mapping is an ill-posed problem and some uncertainty is present. We learn the mapping via a Pix2pix net, also known as conditional GAN (cGAN), an unsupervised generative model which represents a variation of a standard GAN. As such it includes two deep neural networks, a generator *G* and discriminator *D*. The generator aims to capture the data distribution, while the discriminator estimates the probability that a sample actually came from the training data rather than from the generator. In order to learn a generative distribution over the data *x*, the generator builds a mapping *G*(*z*;*θ*_*G*_) from a prior noise distribution *p*_*z*_ to the image data space, *θ*_*G*_ being the generator parameters. The discriminator outputs the probability that *x* came from the real data distribution *p*_*data*_(*x*) rather from the generated one. We denote by *D*(*x*;*θ*_*D*_) the discriminator function, *θ*_*D*_ being the discriminator parameters. In standard GANs, the optimal mappings G* is obtained as the equilibrium point of the min–max game:

$$\left(G^{*},D^{*}\right)=\text{arg}\;\underset{G}{\text{min}}\;\underset{D}{\text{max}}\;L_{GAN}\left(D,G\right),$$

where we have defined the objective function

$$\begin{array}{c} L_{GAN}\left(D,G\right):=E_{x∼p_{data}\left(x\right)}\left[\text{log}\;D\left(x;\theta_{D}\right)\right]+E_{z∼p_{z}\left(z\right)}\left[\text{log}\left(1-D\left(G\left(z;\theta_{G}\right)\right)\right)\right], \end{array}$$ (2)

where E[·] stands for the expected value. In the conditional framework, an extra variable *y* is added as a further source of information on *G*, which combines the noise prior *p*_*z*_(*z*) and *y*. The objective function thus becomes

$$\begin{array}{c} L_{cGAN}\left(D,G\right)=E_{x∼p_{data}\left(x\right)}\left[\text{log}\;D\left(x;\theta_{D}\right)\right]+E_{z∼p_{z}\left(z\right)}\left[\text{log}\left(1-D\left(G\left(z|y;\theta_{G}\right)\right)\right)\right]. \end{array}$$ (3)

**Fig 3:**
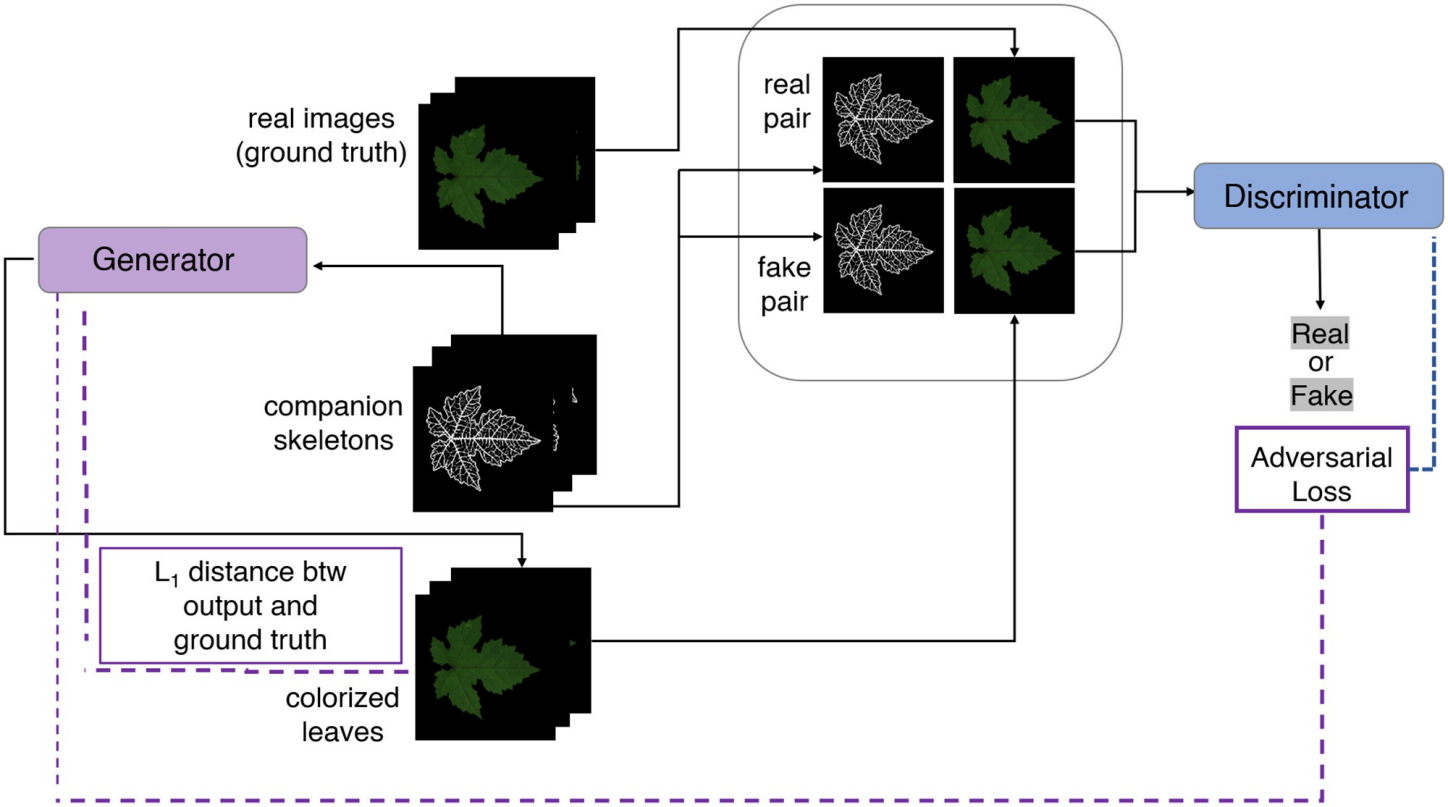
Illustration of the Pix2Pix framework (training).

Previous approaches have found it beneficial to mix the GAN objective with a more traditional loss, such as *L*_2_ distance [27]. The discriminator’s job remains unchanged, but the generator is bound not only to fool the discriminator but also to stay near the ground truth output in an *L*_2_ sense. In this work we rather explore the use of the *L*_1_ distance rather than *L*_2_ as *L*_1_ promotes sparsity and at the same time it encourages less blurring [28]:

$$\begin{array}{c} L_{L_{1}}\left(G\right)=E_{x,y,z}[\|y-G\left(z|y;\theta_{G}\right){\|}_{1}], \end{array}$$ (4)

where we recall that ${\left\|x\right\|}_{1}=\sum_{i=1}^{n}\left|x_{i}\right|$, with *x*_*i*_ begin the *i*–th element of the vector *x*. The final objective is thus

$$\begin{array}{c} \left(G^{*},D^{*}\right)=\text{arg}\;\underset{G}{\text{min}}\;\underset{D}{\text{max}}\;\;L_{cGAN}\left(D,G\right)+\lambda L_{L_{1}}\left(G\right) \end{array}$$ (5)

where λ is a regularization hyperparameter. In our implementation the extra information corresponds to the leaf skeletons which condition *G* in the image generation task to preserve leaf shape and venation pattern. The discriminator is provided with skeleton plus generated image pairs and must determine whether the generated image is a plausible (feature preserving) translation. Fig 3 shows the training process of the cGAN. We refer to Section B in S1 File. for specifications of the Pix2pix architecture we adopted and the relative training strategy.

#### L2L workflow: From random samples to leaf images

**Fig 4:**
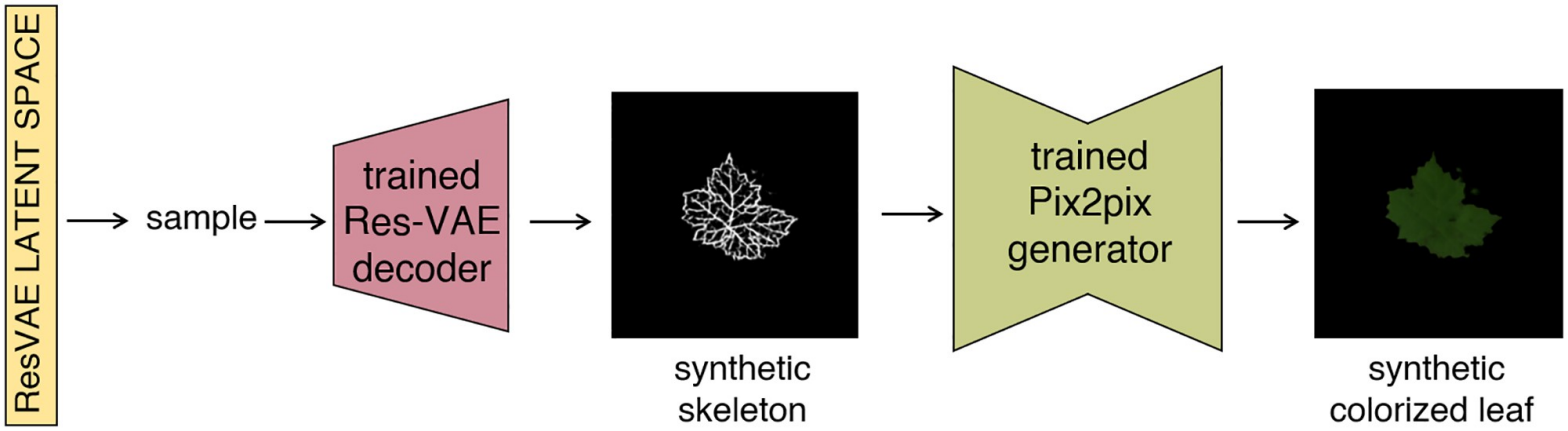
L2L workflow illustration. A random input vector is drawn from the ResVAE latent space representation and is input into the trained ResVAE decoder. This latter outputs a synthetic leaf skeleton, which in turn is fed into the trained generator of the Pix2Pix and translated into a corresponding colorized leaf.

Upon training of the ResVAE and Pix2pix architectures, we dispose of an end-to-end procedure for the generation of synthetic leaves. The procedure, which is completely unsupervised, can be summarized as follows (see also Fig 4):

1. Load weights of the trained ResVAE decoder and Pix2pix generator.
2. Draw a random vector from a normal distribution whose parameters are chosen according to the ResVAE latent space representation (note that its size equals the dimension of the latent space used in the ResVAE, 32 in the present case).
3. Input the random vector in the trained ResVAE decoder and generate a leaf skeleton
4. Input the leaf skeleton into the trained generator of the Pix2Pix net to translate it into a fully colorized leaf.

## Results

The proposed technique can be employed to generate as many synthetic leaf images as the user requires. The model has been implemented with Keras. The code and data for this project are available on GitHub at https://github.com/AleBenfe/Leaf2Leaf. Upon generation of the synthetic images, their quality is assessed performing both qualitative (visual) and quantitative evaluations as detailed here below.

### Visual qualitative evaluation

#### Consistency test

Beforehand, we have evaluated the consistency of the methodology by verifying that the net has learned to translate a leaf sample comprised in the training set into itself.

#### Translation from unseen real companion skeleton

**Fig 5:**
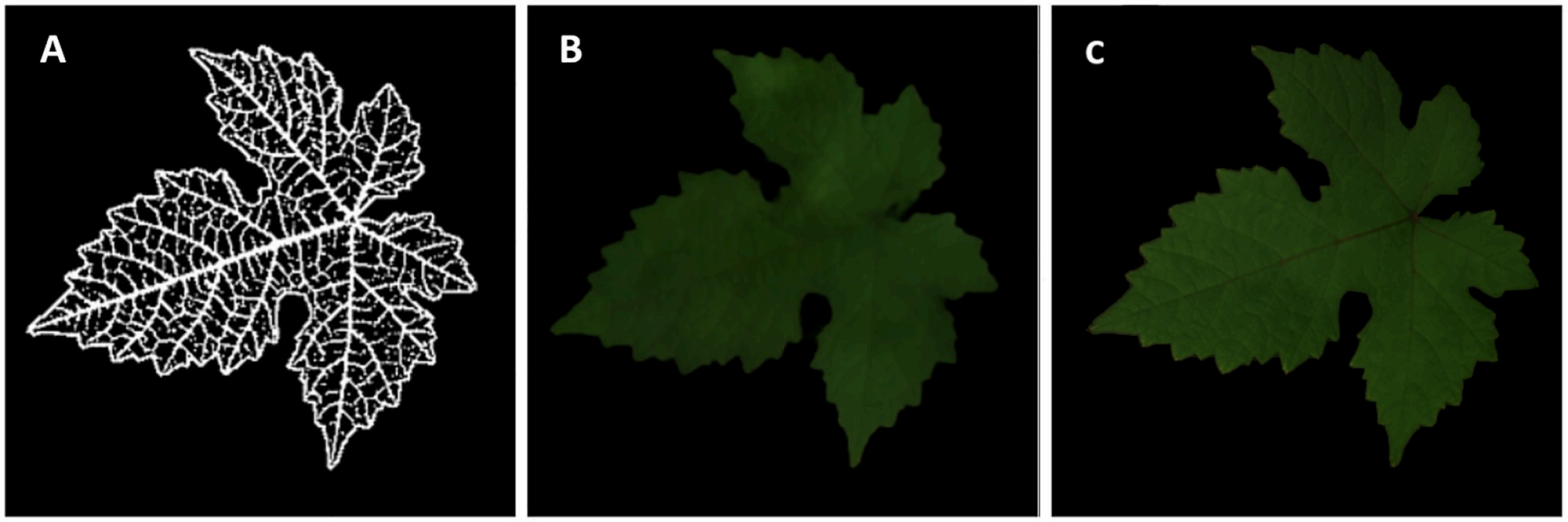
Translation from unseen real companion skeleton. A binarized leaf skeleton companion of a real leaf not belonging to the training set is passed through the generator of the Pix2Pix net to check. A: companion skeleton; B: synthetic colorized blade; C: real image.

We verify that it is able to produce reliable synthetic images using skeletons obtained from leaves that are not part of the training dataset. Fig 5 shows an instance of colorized leaf obtained from this test: the overall quality is highly reliable, except for small vein discoloration and a small blurring effect, which is a well–known product of AEs employed in image generation [29].

#### Full L2L translation

**Fig 6:**
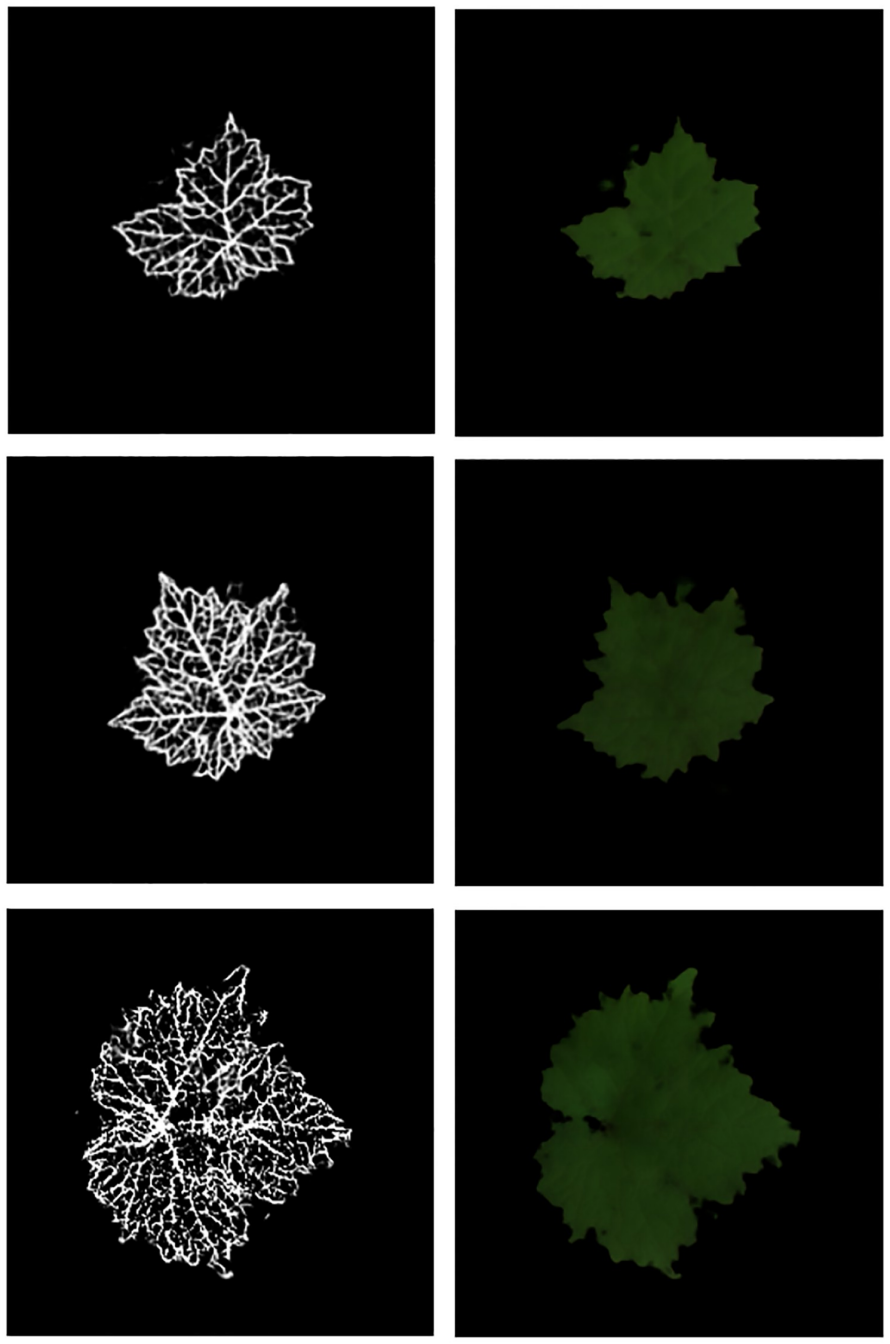
Full L2L translation results. Examples of synthetic colorized leaves along with the corresponding synthetic companion skeletons.

Fig 6 shows several instances of synthetic colorized leaves obtained starting from different random latent vectors. Note that the generated leaf images differ in terms of their global appearance, that is the model generalizes and does not trivially memorizes the examples. As a note, one should observe that some discolored parts may be appear. Moreover, sometimes the skeletons show small artifacts consisting in not–connected pixels positioned outside the leaf boundary (not appearing in Fig 6). This latter issue will be addressed via a refinement algorithm explained below.

#### L2L-RGNIR translation

**Fig 7:**
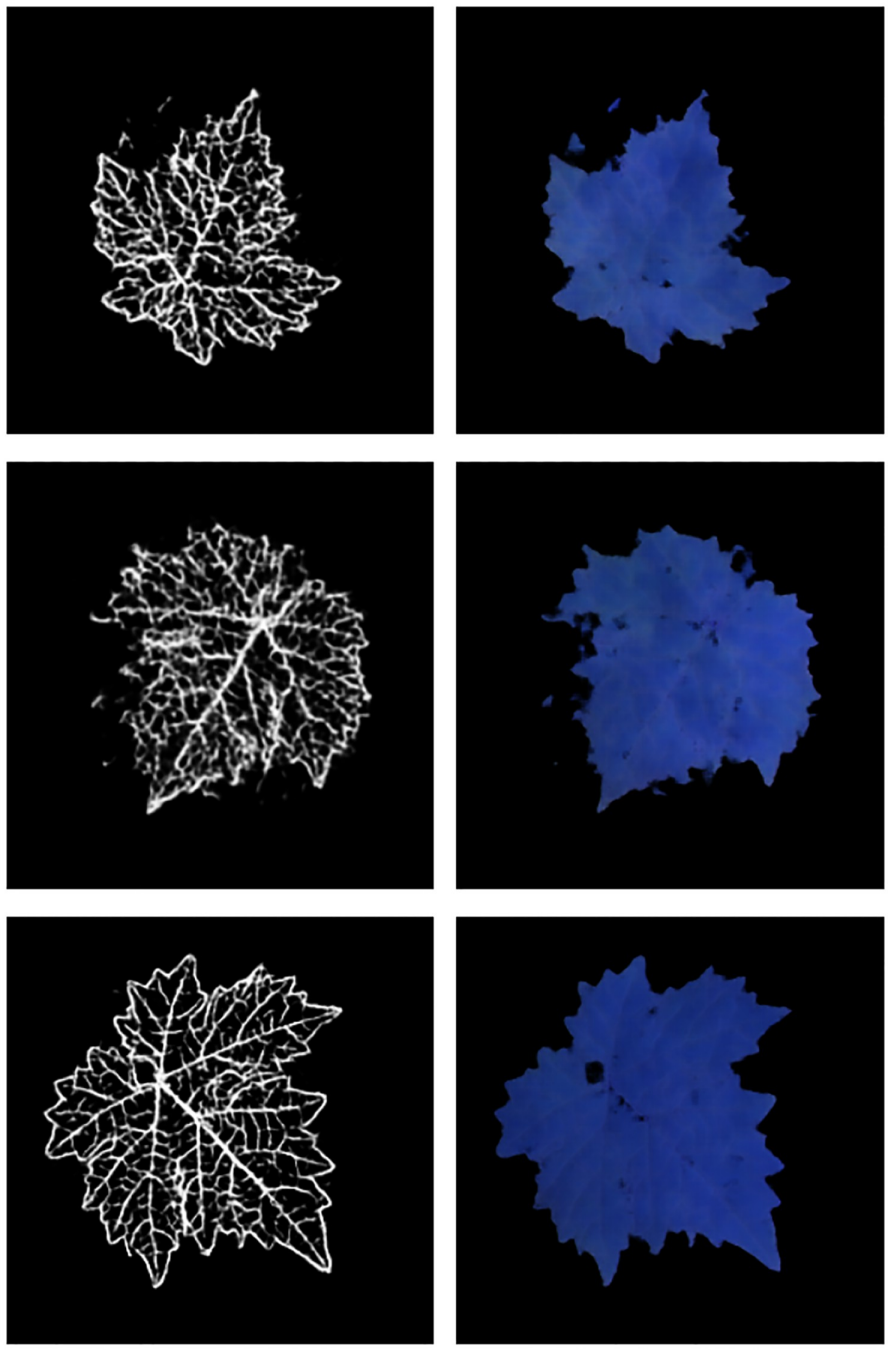
L2L-RGNIR translation results. Examples of synthetic leaves colorized in the RGNIR channels along with the corresponding synthetic companion skeletons.

As mentioned above, applications in crop management require to have at disposal images also in the NIR channel. To do this, we use the L2L generation procedure as for the RGB channels starting from RGNIR images as Pix2Pix targets, using the same approach without modifications. Since the same leaf skeletons are used, it is not necessary to re-train the ResVAE if this procedure has been already carried out for the RGB case. Fig 7 shows some results of this model.

#### Refinement algorithm

**Fig 8:**
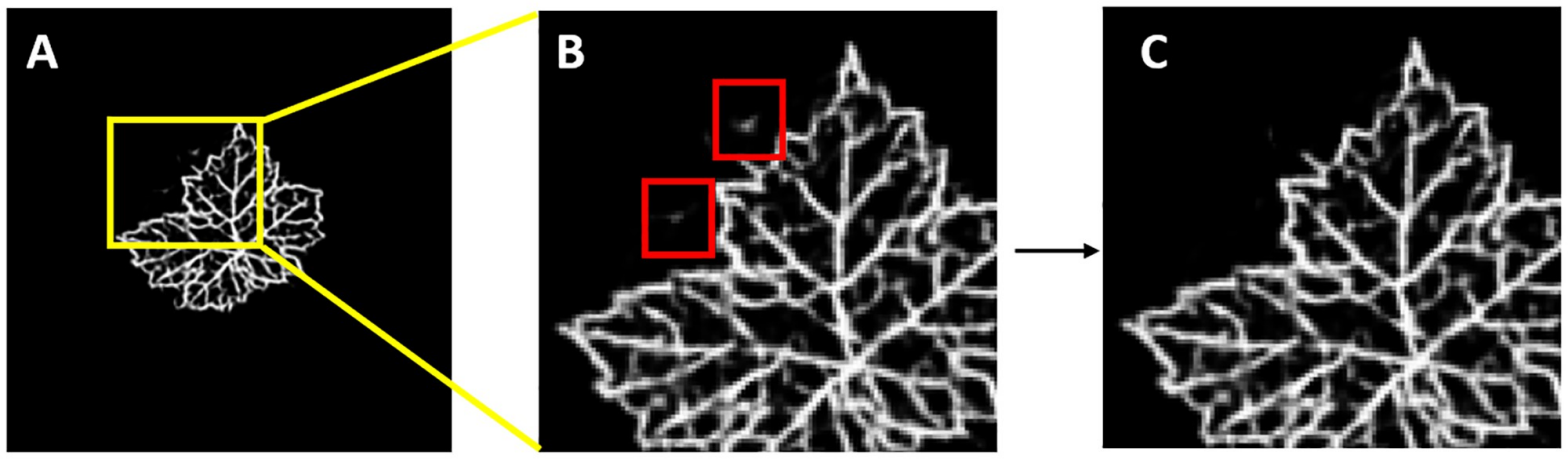
Refinement algorithm. The generative procedure sometimes produces artifacts, that is leaf regions that appear outside the leaf blade. These artifacts are corrected by procedurally finding the contours of all the objects in the image and removing the objects outside the leaf contour. A: first leaf in Fig 6 presenting artifacts; B: inset showing the magnified artifacts; C: cleaned leaf.

We have already discussed the fact that synthetically generated images may sometimes present artifacts (leaf regions that appear detached from the leaf blade). Obviously this is not realistic and we need to remove such artifacts. The refinement algorithm is implemented at present in a procedural way and it is based on the idea of finding the contours of all the objects and removing all objects laying outside the leaf contour. Note that this procedure must pay attention to leave internal holes intact, because in nature such holes are the result of the superposition of leaf lobes or due to several abiotic/biotic conditions. Fig 8 shows the first leaf in Fig 6 which presents artifacts (panel A, zoomed area including the artifact in panel B) and its cleaned counterpart (panel C).

### Quantitative quality evaluation

**Fig 9:**
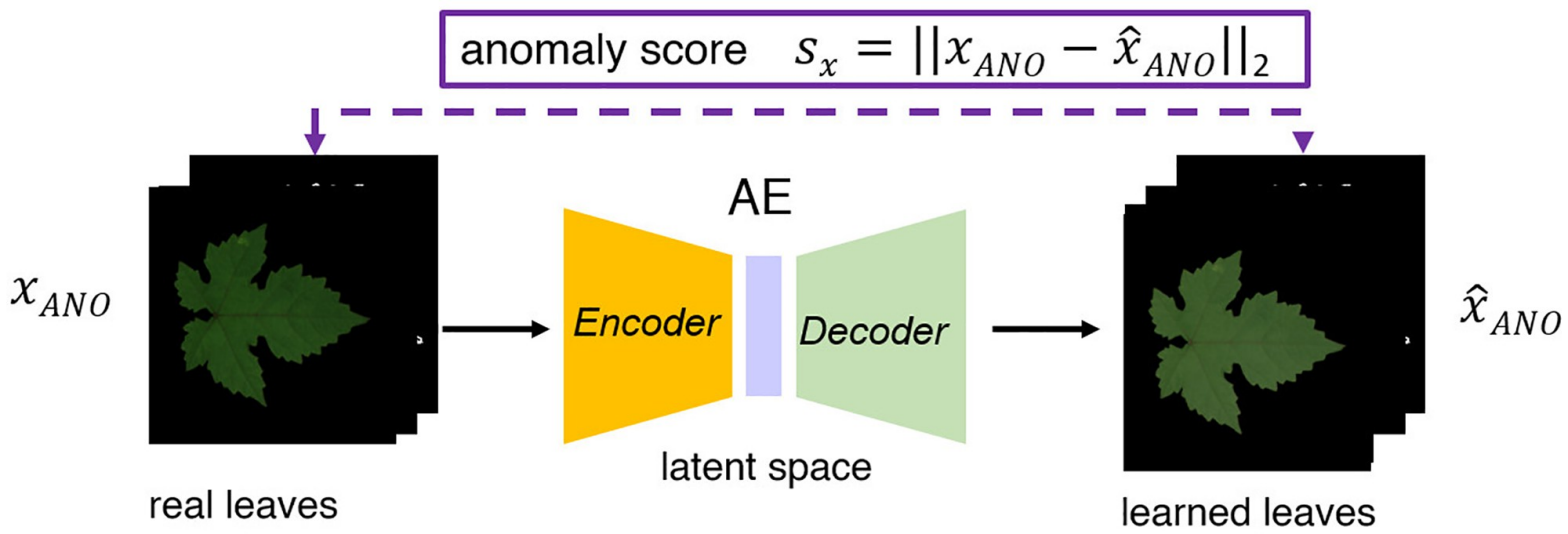
AE for anomaly detection. The AE is trained with images of real leaves to be the identity operator of the input. A synthetic leaf with a low level of similarity is recognized as an anomaly if fed into the trained AE and its anomaly score *s*_*x*_ is high.

In order to assess quantitatively the quality of the generated leaves, we employ a DL–based anomaly detection strategy. This approach is discussed in detail in [30], here we briefly recall the main points. The strategy consists in training an AE to compress *real* leaf images in a latent subspace and then reconstruct the images using the latent representation (see Generative methods for L2L translation section for the same concept). Once the network is trained in this way, we feed it with a synthetic image generated by our procedure. The AE encodes it in the latent space and tries to recover the original image according to its training rules. Since the net has been trained to be the identity operator for real images, if the artificial images are substantially different, an anomalous reconstruction is obtained. Fig 9 provides a visual schematization of this approach. The figure also details the score system used to detect the anomaly.

**Fig 10:**
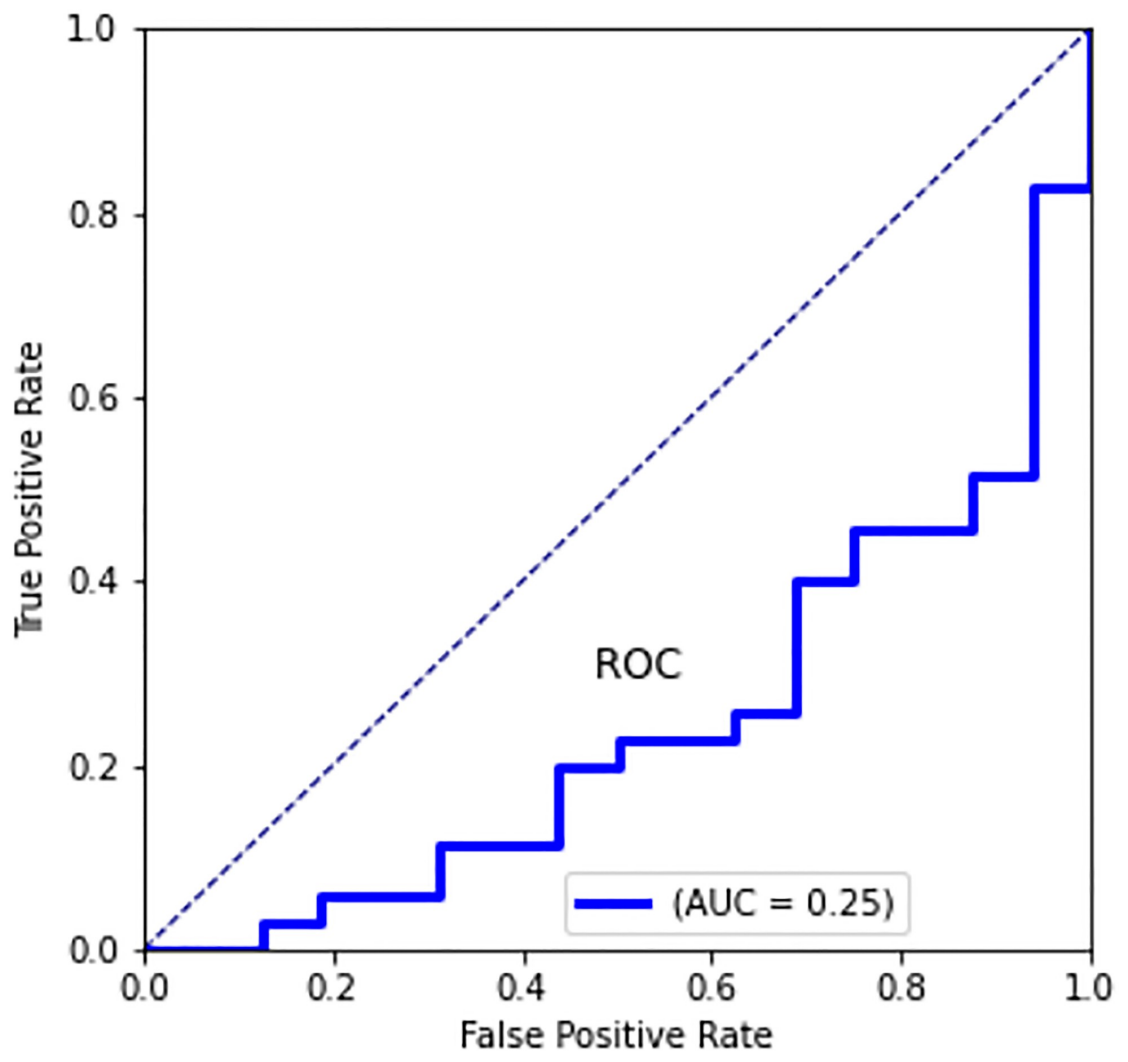
Quantification of anomaly via ROC curve and AUC index. A point on the ROC curve represents—for a certain threshold on the anomaly score—the *FPR* vs the *TPR* as defined in (6). We found AUC = 0.25, which means that a synthetic image is classified as synthetic in the 25% of cases and in the 75% is considered real. The dotted line represents the result one would obtain by tossing a coin to decide whether an image is artificial or real.

The degree of anomaly is quantified via the Receiver Operating Characteristic (ROC) curve and the corresponding Area Under Curve (AUC) index [31]. A point on the ROC curve represents the False Positive Rate (FPR, *x*-axis) vs the True Positive Rate (TPR, synonim of recall, *y*-axis) for a certain threshold of the anomaly score, *FPR* and *TPR* being defined in this context as

$$\begin{array}{c} FPR=\frac{FP}{FP+TN},\;TPR=\frac{TP}{TP+FN} \end{array}$$ (6)

where *TN* = no. of genuinely real images correctly classified as real, *TP* = no. of genuinely fake images correctly classified as fake, *FN* = no. of genuinely fake images incorrectly classified as real and *FP* = no. of genuinely real images incorrectly classified as fake. The looser the criteria for determining a positive result, the more points on the curve move upward and to the right since more items are classified as positive (thus increasing both FPs and TPs). The AUC is the value of the 2D area under the ROC curve and provides an aggregate measure of performance across all possible classification thresholds. We found AUC = 0.25: this results can be interpreted as the fact that, for a random synthetic (and unseen) image fed into the AE, there is a 25% of possibility to classify it as an anomaly, that is to be synthetic instead of real. The results are shown in Fig 10. While we do not attain a perfect generation of synthetic leaves, these results show that they are a reasonably accurate surrogate of real leaves and can be used for a first massive training at a very low cost. A successive refinement can then be applied using a limited number of real leaves upon transfer–learning techniques.

### Application to image-based sensing of plants: Early recognition of powdery mildew symptoms on cucumber leaves

**Fig 11:**
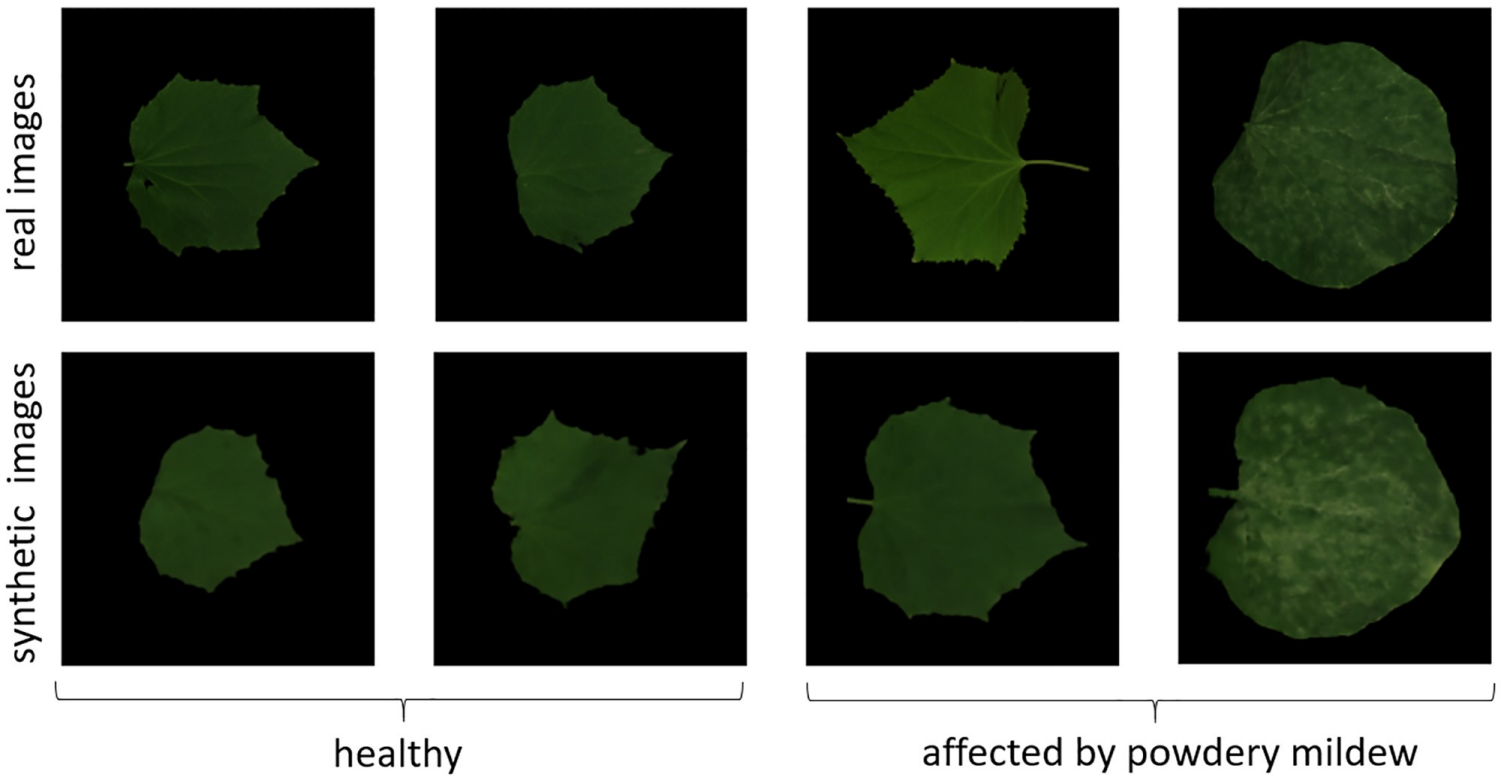
Real and synthetic images of cucumber leaves. Real images were acquired with the procedure described in the Material and Methods section, synthetic images were generated via the L2L algorithm. Diseased leaves are affected by powdery mildew with different severity levels: the whitish spots on the leaf blade are signs of early-to-mid powdery mildew infection. Notice that while advanced signs of podwery mildew are easily recognizable, early stages signs are much more elusive.

As an illustrative example of the proposed methodology, we consider an application in image-based sensing of plants, an advanced crop-management approach aimed at supporting and maximizing efficiency of farming processes [32]. Given the enormous volume and the heterogeneity of the data generated by these systems, automatic image-analysis for monitoring the status of the plants plays a key-enabling role. To this aim, the applications of deep learning methods based on CNNs in this domain have recently dramatically expanded [33]. Specifically, here we consider the task of training a neural network to automatically detect early symptoms of powdery mildew (a major fungal disease which mostly affects leaves in many crop plants, exhibiting common symptoms as whitish spots on the leaf blade tissue) in images of cucumber *Cucumis sativus* leaves. To do this, one should collect a sufficiently large and well–balanced dataset of both healthy and diseased leaves. In order to enrich the available datasets of real leaves, we generated two datasets of synthetic images with the algorithm presented above, a set of healthy leaves and one of leaves affected by powdery mildew with different severity levels. To produce the datasets, we started from collections of images of real healthy and diseased cucumber leaves acquired with the same modalities described in the Material and Methods section (see [30] for a detailed description of these collections). Fig 11 shows examples of real and synthetic (generated via the L2L algorithm) images of healthy and diseased leaves.

**Fig 12:**
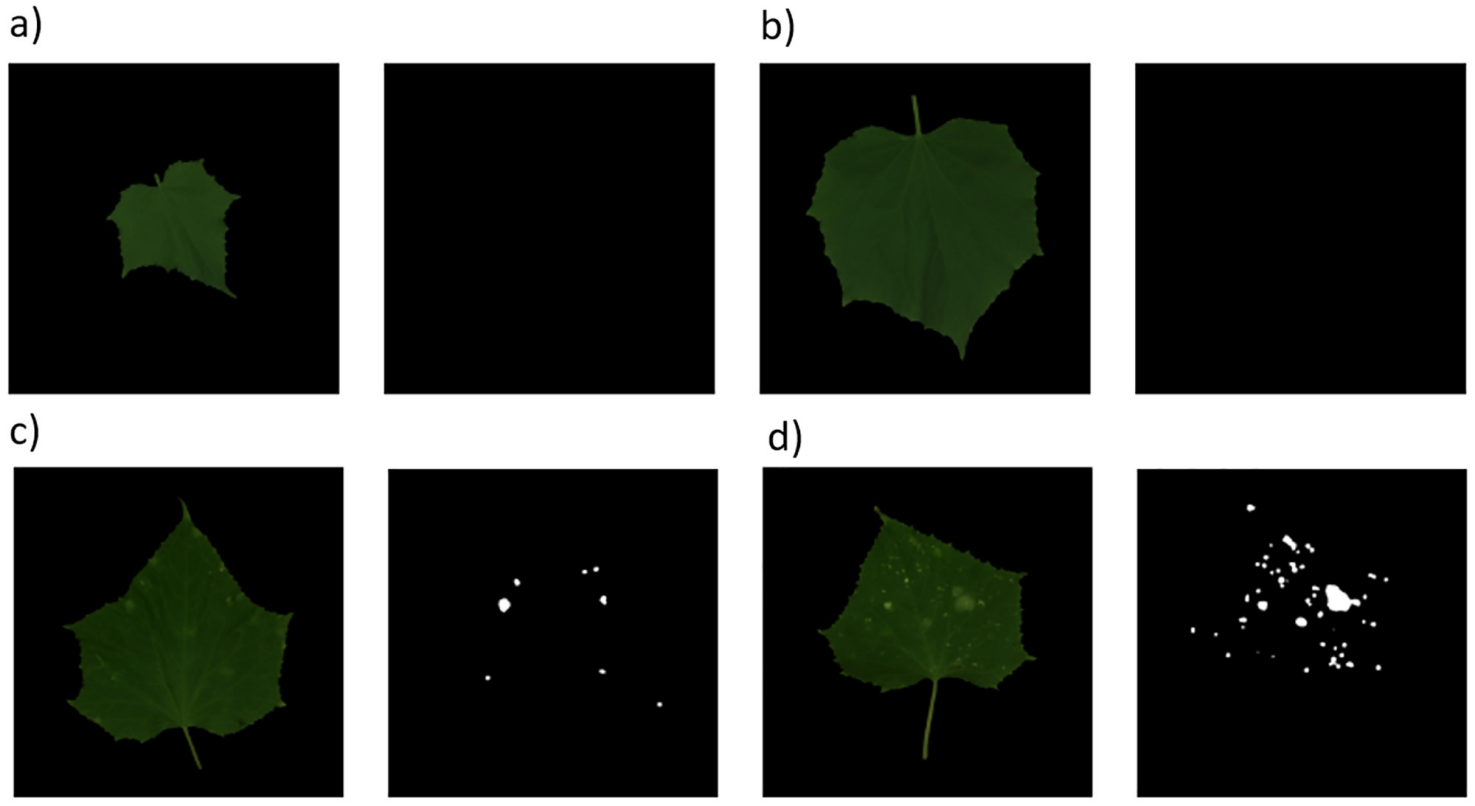
Segmentation masks. The masks, which are produced by a U-net architecture trained with a mix of real and synthetic leaves, denote the diseased spots. The masks are empty for the healthy leaves (a-b), while they indicate disease spots with an accuracy above 94%—according to the segmentation of a human expert—for leaves affected by powdery mildew (c-d).

We use supervised U-net architectures (*e.g.*, see [34]) trained with a mix of real and synthetic leaves from the healthy and diseased collections to perform semantic segmentation. Without the addition of synthetic leaves, we were not able to perform the training, since we disposed of too few samples. Fig 12 (right column) shows the segmentation masks (indicating diseased regions) obtained from real healthy (a-b) and diseased (c-d) test images—*i.e.* never seen by the net—of leaves. The U-net produces empty masks for healthy leaves since no disease spots have been recognized, while it correctly recognizes (with more than 94% accuracy) powdery mildew spots on diseased leaves (c-d). The correctness was validated by an expert plant pathologist.

## Discussion and conclusions

Goal of this work was to explore advanced DL generative methods to produce synthetic images of leaves to be used in computer–aided applications. In these latter fields, several approaches for the generation of synthetic leaves do exist in literature, but, the best of our knowledge, this is the first attempt to use a DL-based generative methodology. The main focus was on the generation of artificial samples of leaves to be used to train DL networks for modern crop management systems in precision agriculture. The L2L procedure took inspiration from works aimed at the generation of synthetic images of the fundus of the eye, which is typically composed of a tissue background on which retinal vessels are superposed. However, leaves generally show more complexity and variation patterns and algorithms had to be adapted to our specific context. Positive impacts of the present work are:

- the availability of synthetic samples which have a *quantitive*—and not only qualitative—resemblance to real leaf sample so to alleviate the burden of manually collecting and annotating hundreds of data
- image-sensing in smart agriculture is an innovative approach which can deeply impact everybody’s life, being connected to effective and sustainable food production. We have discussed an application of the present algorithm to this context, which we believe can lead to a significant improvements in the use of these technologies
- we originally proposed the use of an AnoGAN to provide a quantitative evaluation of the results, which remains a delicate point
- while our target application was the enrichment of datasets for DL-based image sensing in smart agriculture, we deem that the present approach may be of interest also in a wider range of collateral fields, including computer graphics applications in all their declinations.

The generated images show a certain number of defects. We have observed that the critical part is the generation of a correct skeleton. A quality check on the quality of this latter can filter out skeletons that do not represent the typical structure of a certain leaf (observe that this structure can be strongly dependent on the leaf species). Once one has obtained a plausible skeleton, the Pix2pix net performs good translations from the leaf skeletons generated by the ResVAE, except for some discolored parts, both for the colorization of RGB and RGNIR images. Also, the leaves generated by ResVAE have sometimes pixels positioned outside the boundary which, if not corrected, can cause artifacts in the synthetic leaves. Recognizing and correcting these artifacts can be implemented in an easy procedure. In our work we resized images to a relatively low resolution. Available GANs, as StyleGANs, are indeed able to generate images with a higher resolution. However, these architectures require a very large (of the order of many thousands) train dataset in order to achieve good results. The point of the present work is rather to explore the feasibility of the approach and its validity for our target application. The general approach we propose does not really depend on the specific generative architecture and other generalist architectures could be used as well. What we instead find more interesting are the specific observations relative to the generations of leaves, that is the strong importance of a good skeleton in order to obtain quantitatively correct results (and not only a qualitatively reasonably correct appearance). Eventually, we observe that several computer–aided applications may also benefit of such a strategy, where many samples are required, possibly with different degree of accuracy in the representation. This is especially true in all applications related to medicine, where the availability of data is a crucial point.

## Supporting information

S1 File Implementation and training of the neural architectures. This SI file provides details about the ResVAE and Pix2pix net architectures along with the respective training strategies.
(PDF)

S2 File Why a two-step generative approach?. This SI file provides motivation of the choice of organizing synthetic leaf generation into a two step procedure.
(PDF)
