# Supplementary material for: A deep learning generative model approach for image synthesis of plant leaves
Source: PLoS One. 2022 Nov 18;17(11):e0276972. doi: 10.1371/journal.pone.0276972 (PMC9674145; doi:10.1371/journal.pone.0276972)
Supplement: S1 File — This SI file provides details about the ResVAE and Pix2pix net architectures along with the respective training strategies. (PDF) [file pone.0276972.s001.pdf]

# S1 Appendix. Implementation and training of the neural architectures.

## A) Implementation and training of the ResVAE neural network.

The architecture, inspired by the one described in [1], is shown in Fig 1.

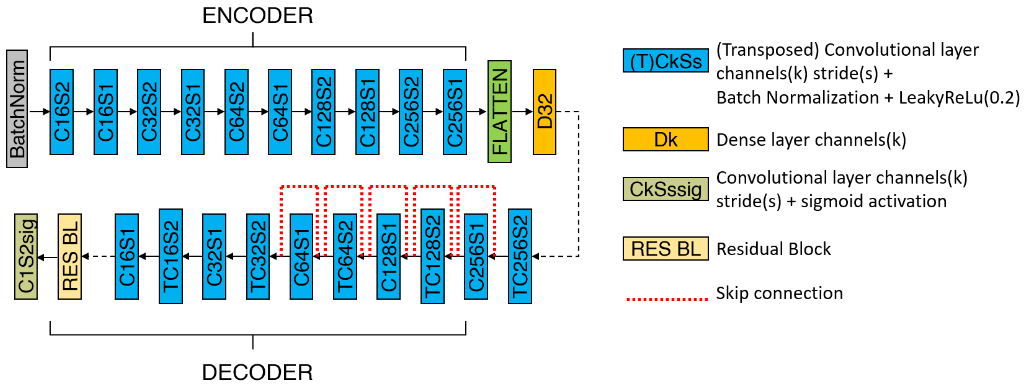


Figure 1: **ResVAE architecture.** Building blocks of the encoder and decoder components of the ResVAE. The convolutional filters have kernels of size $4 \times 4$. The Residual block is formed by 5 convolutional layers of 16 filters each with kernel of size $4 \times 4$ and stride equal to 1, followed by a Batch Normalization layer and LeakyReLU activation function.

The training is performed via a stochastic gradient descent strategy, with gradients computed by standard back–propagation; we use the ADAM optimizer with learning rate $\eta = 0.001$ and we train the model for 2000 epochs with a batch size of 64. After a hyper–parameter search, $\beta$ in the loss function

$$\mathcal{L}_{\text{VAE}}(x, \hat{x}) = \mathcal{L}_{L_2}(x, \hat{x}) + \beta \mathcal{L}_{KL}\left(p(z|x), \mathcal{N}(0,1)\right) \tag{1}$$

was set to 75.

## B) Implementation and training of the Pix2pix neural network.

The Pix2pix net is a GAN architecture designed for image-to-image translation, originally presented in [2] and comprising a generator and a discriminator. The discriminator is deep neural network that performs image classification. It takes both the source image (leaf skeleton) and the target image (colorized leaf) as input and predicts the likelihood of whether the target image is real or a fake translation of the source image. We use a PatchGAN model which tries to establish whether each $N \times N$ (local) patch in the image is real or fake. We run this discriminator convolutionally across the image, averaging all responses to provide the ultimate output of the discriminator. The generator is an encoder-decoder model using a U-Net architecture with feature-map concatenation between two corresponding blocks of the encoder/decoder. The encoder and decoder of the generator are comprised of standardized blocks of convolution, batch normalization, dropout, and activation layers. We proceed as suggested in [2]: the generator is updated via a weighted sum of both the adversarial loss and the $L_1$ loss, where the parameter $\lambda$ in the loss function in Equation (**??**) is set to 100 in order to encourage the generator to produce plausible translations of the input image, and not just plausible images in the target domain. We initialize the generator/discriminator weights with a normal distribution of zero mean and standard deviation $\sigma = 0.002$; we use the ADAM optimizer with a learning rate $\eta = 0.0002$

and we train the generator/discriminator paired model for 12000 training steps, using a batch size of 1. Fig 2 shows the generator and discriminator architectures.

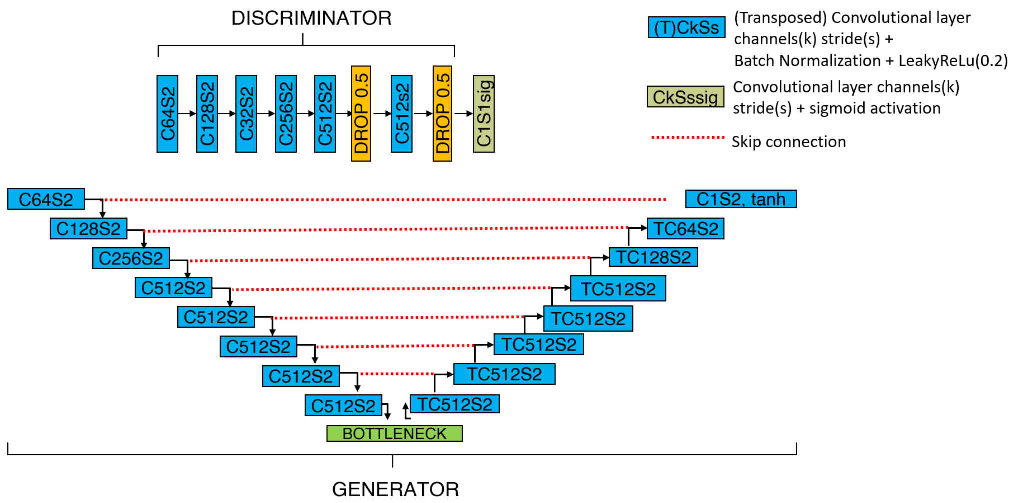


Figure 2: `Pix2pix` **architecture.** Building blocks of the generator and discriminator components.

# References


[1] Chollet F. Variational AutoEncoder; 2020. Web site: https://keras.io/examples/generative/vae/.

[2] Isola P, Zhu JY, Zhou T, Efros AA. Image-to-image translation with conditional adversarial networks. In: Proceedings of the IEEE conference on computer vision and pattern recognition; 2017. p. 1125–1134.
