# Supplementary material for: A deep learning generative model approach for image synthesis of plant leaves
Source: PLoS One. 2022 Nov 18;17(11):e0276972. doi: 10.1371/journal.pone.0276972 (PMC9674145; doi:10.1371/journal.pone.0276972)
Supplement: S2 File — This SI file provides motivation of the choice of organizing synthetic leaf generation into a two step procedure. (PDF) [file pone.0276972.s002.pdf]

S2 Appendix. Why a two-step generative approach?

The concept of the present work takes inspiration from the synthetic generation of eye fundus images for medical applications. In such a field, it is a consolidated procedure to split the generation process into: *i)* the generation of the skeleton; *ii)* colorization of the fundus image (*e.g.*, see [1] and also [2] from a different research group). In our opinion, this is related to applications where a very low number of images are available for training, so that the generation of the skeleton, which is vital to obtain a realistic image, is decoupled and separately addressed and controlled. Notice that the number of images we have at our disposal is comparable to those of these medical-related literature, being in the order of hundreds of images. In Fig 1 we present some earlier results of us, where we used a one-step procedure. It is apparent that these images are very blurred and leaf veins are completely lacking.

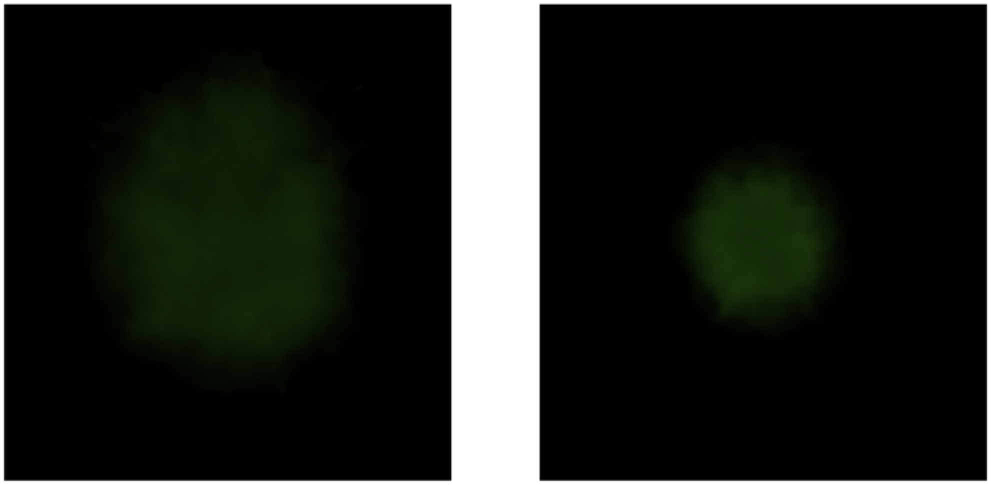

Figure 1: **Results of one-step procedure.** When generating images of synthetic leaves, we have found to be much more effective to handle separately the vein skeleton and the colorization of the leaf blade. Here we show two earlier attempts, where we used a one-step procedure. The results are very blurred and veins are practically non–existent.

References

[1] Costa P, Galdran A, Meyer MI, Niemeijer M, Abràmoff M, Mendonça AM, et al. End-to-end adversarial retinal image synthesis. IEEE Transactions on Medical Imaging. 2017;37(3):781–791.

[2] Zhao H, Li H, Maurer-Stroh S, Cheng L. Synthesizing retinal and neuronal images with generative adversarial nets. Medical image analysis. 2018;49:14–26.
